# The role of tRNA-Derived small RNAs (tsRNAs) in pancreatic cancer and acute pancreatitis

**DOI:** 10.1016/j.ncrna.2024.12.011

**Published:** 2024-12-30

**Authors:** Yan Pan, Xiaowei Ying, Xueting Zhang, Hongting Jiang, Junjie Yan, Shiwei Duan

**Affiliations:** a Department of Integrative Oncology, The First People's Hospital of Fuyang, Fuyang First Hospital Affiliated to Zhejiang Chinese Medical University, Hangzhou, China; b Department of Clinical Medicine, School of Medicine, Hangzhou City University, Hangzhou, Zhejiang, China

**Keywords:** tsRNAs, Pancreatic cancer, Acute pancreatitis, Gene regulation, Biomarkers

## Abstract

tRNA-derived small RNAs (tsRNAs), encompassing tRNA fragments (tRFs) and tRNA-derived stress-induced RNAs (tiRNAs), represent a category of non-coding small RNAs (sncRNAs) that are increasingly recognized for their diverse biological functions. These functions include gene silencing, ribosome biogenesis, retrotransposition, and epigenetics. tsRNAs have been identified as key players in the progression of various tumors, yet their specific roles in pancreatic cancer (PC) and acute pancreatitis (AP) remain largely unexplored. Pancreatic cancer, particularly pancreatic ductal adenocarcinoma, is notorious for its high mortality rate and extremely low patient survival rate, primarily due to challenges in early diagnosis. Similarly, acute pancreatitis is a complex and significant disease. This article reviews the roles of 18 tsRNAs in PC and AP, focusing on their mechanisms of action and potential clinical applications in these two diseases. These tsRNAs influence the progression of pancreatic cancer and acute pancreatitis by modulating various pathways, including ZBP1/NLRP3, Hippo, PI3K/AKT, glycolysis/gluconeogenesis, and Wnt signaling. Notably, the dysregulation of tsRNAs is closely linked to critical clinical factors in pancreatic cancer and acute pancreatitis, such as lymph node metastasis, tumor-node-metastasis (TNM) stage, overall survival (OS), and disease-free survival (DFS). This article not only elucidates the mechanisms by which tsRNAs affect pancreatic cancer and acute pancreatitis but also explores their potential as biomarkers and therapeutic targets for pancreatic cancer. The insights provided here offer valuable references for future research, highlighting the importance of tsRNAs in the diagnosis and treatment of these challenging diseases.

## 1 Introduction

**Fig. 1:**
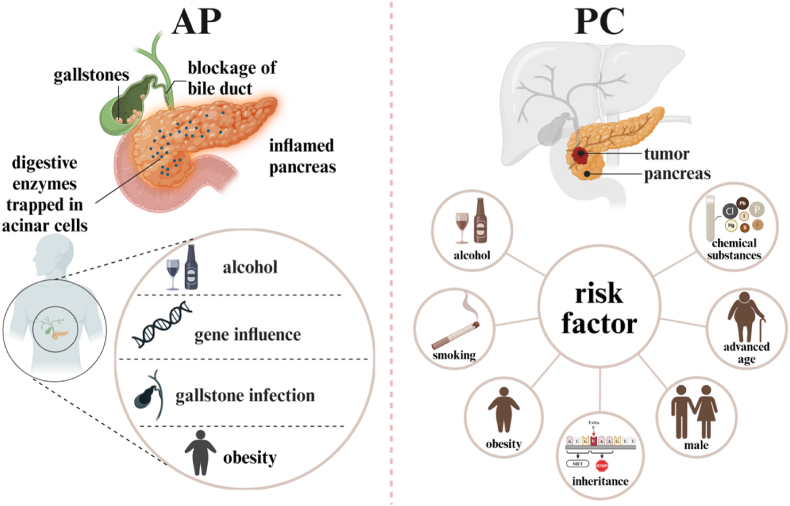
Introduction to pancreatic related diseases. On the left is an introduction to AP, where a blockage in the cystic duct connected to the gallbladder traps digestive enzymes within the acinar cells, causing inflammation. AP can be triggered by factors such as alcohol consumption, genetic predispositions, gallbladder infections and obesity. The disease has a mortality rate of around 15 %, with its incidence steadily rising. On the right, risk factors for PC include alcohol consumption, smoking, obesity, genetic predispositions, male, advanced age, and exposure to chemicals. AP, acute pancreatitis; PC, Pancreatic cancer.

The pancreas is a vital organ with both exocrine and endocrine functions, crucial for food digestion and blood sugar regulation. Impaired pancreatic function can lead to severe diseases such as pancreatic cancer, acute pancreatitis, chronic pancreatitis, and diabetes, affecting over 10 % of the global population [1]. Pancreatic cancer, particularly pancreatic ductal adenocarcinoma (PDAC), is a major concern as it has become one of the leading causes of cancer death worldwide [1]. PDAC is the seventh leading cause of cancer death globally, with the highest mortality rate among all solid tumors [2]. PC is one of the most common malignant tumors of the digestive system, with approximately 90 % originating from the glandular epithelium of the pancreas. The most frequent site of occurrence is the pancreatic head [3,4]. The incidence of pancreatic cancer has risen in recent years, representing 2 % of all cancers and causing about 5 % of cancer-related deaths. While the pathogenesis of pancreatic cancer remains incompletely understood, studies suggest that long-term smoking, alcohol consumption, obesity, male sex, advanced age, and exposure to certain chemicals may increase its risk (Fig. 1). Additionally, genetic predisposition plays a critical role, with individuals who have a family history of the disease facing a significantly higher risk [5] (Fig. 1). Early symptoms are often absent, with approximately 90 % of patients showing no obvious symptoms and 10 % having a familial genetic predisposition [6]. The five-year survival rate is alarmingly low at about 4 %, primarily due to late-stage diagnosis in 80–85 % of cases, precluding surgical intervention [6]. Despite ongoing research, survival rates have not significantly improved, and incidence rates continue to rise [7](Fig. 1).

Acute pancreatitis (AP) is another prevalent pancreatic disease, characterized by inflammation of the exocrine pancreas, severe abdominal pain, and multiple organ dysfunction. Under normal conditions, digestive enzymes in pancreatic acinar cells are stored as inactive zymogens. When triggered by pathogenic factors such as alcohol or drugs, these zymogens become activated within the acinar cells, directly causing cellular damage and resulting in AP [8,9] (Fig. 1). Bile duct obstruction, particularly due to cholelithiasis, is another major cause of AP. Obstruction caused by gallstones, roundworms, or other factors can prevent bile from entering the duodenum, leading to bile reflux into the pancreatic duct. This reflux activates digestive zymogens within the duct, initiating the pancreas's autodigestion process and triggering AP [10,11]. Genetic predisposition and obesity are additional factors contributing to the onset of AP [12] (Fig. 1). Severe cases can result in pancreatic necrosis and persistent organ failure, with mortality rates reaching up to 15 %. The global incidence of AP is increasing, with approximately 30–40 new cases per 100,000 people annually [13]. AP can cause long-term health issues such as chronic weakness, recurrent episodes, and pancreatic insufficiency (Fig. 1).

tRNA-derived small RNAs (tsRNAs), including tRNA fragments (tRFs) and tRNA-derived stress-induced RNAs (tiRNAs), have emerged as significant regulators in various biological processes [14]. These RNA fragments are produced through precise cleavage by specific ribonucleases and are classified based on their length and physiological function [[15], [16], [17], [18]]. tsRNAs play critical roles in gene expression regulation, ribosome biogenesis, retrotransposition, and epigenetics, and their dysregulation is closely associated with disease progression [19,20].

The study of tsRNAs is particularly important for the diagnosis and treatment of pancreatic cancer. By understanding the mechanisms through which tsRNAs influence pancreatic cancer, new therapeutic avenues can be explored, potentially improving early detection, precision treatment, and prognosis management for patients.

## 2 tsRNA

**Fig. 2:**
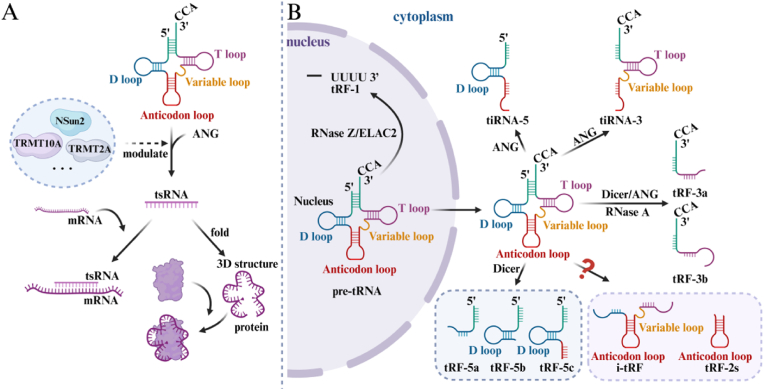
Modification, Classification and Sources of tsRNA. NSun2, NOP2/Sun RNA methyltransferase family member 2; TRMT10A, tRNA methyltransferase 10 homolog A; TRMT2A, tRNA methyltransferase 2 homolog A; mRNA, messenger RNA; tsRNA, tRNA-derived small RNA; pre-tRNA, precursor-tRNA; tiRNA, tRNA-derived stress-induced RNAs; tRF, tRNA-derived fragments; i-tRF, internal tRF; ELAC2, ELAC protein 2; ANG, angiotensin.

Traditionally, the function of tsRNAs is mediated by Argonaute family proteins to achieve RNA silencing. However, recent studies have shown that tsRNAs can form aptamer RNAs that bind to specific targets, creating functional ribonucleoprotein (RNP) complexes [21,22](Fig. 2A).

The modification state of tRNA significantly affects tsRNA production, particularly through methylation and demethylation processes. Methyltransferases such as NSun2, TRMT2A, and TRMT10A influence ANG-mediated tiRNA production [[23], [24], [25], [26]]. For example, TRMT2A specifically methylates the uridine at position 54 in tRNA. Silencing TRMT2A reduces the modification at the U54 position, thereby triggering tsRNA production [24]. Additionally, queuosine modification protects tRNA from cleavage, thus inhibiting tsRNA production [27]. tRNA modifications not only affect tsRNA biosynthesis but may also alter their function. In mature sperm, modifications enhance tsRNA stability and transgenerational effects [28,29](Fig. 2A).

High-throughput RNA sequencing technology has advanced tsRNA discovery, but the highly modified nature of tsRNAs presents challenges. Emerging technologies like PANDORA-seq, which remove key modifications, offer reliable solutions for tsRNA sequencing [30]. Furthermore, tsRNA annotation pipelines such as SPORTS1.0 support tsRNA research [31]. As a novel type of non-coding RNA, tsRNAs exhibit high tissue specificity and are associated with various diseases [32,33]. tsRNAs influence disease progression by regulating translation, transcription, or post-transcriptional processes, playing a crucial role in the tumor microenvironment and drug resistance regulation [34,35]. The stable expression of tsRNAs makes them potential biomarkers for diagnosis and prognosis [35].

## 3 Types of tRNA-Derived small RNAs

### 3.1 tRNA-derived fragments (tRFs)

tRNA-derived fragments (tRFs) are products formed when pre-tRNA or mature tRNA is cleaved by enzymes such as RNaseZ, RNase A, Dicer, and ELAC2. These fragments possess structural characteristics including a 5′ phosphate group and a 3′ hydroxyl group [36](Fig. 2B). tRFs range from 14 to 30 nucleotides in length and function similarly to miRNAs, exerting significant regulatory effects [37]. Based on their location within tRNA transcripts, tRFs are categorized into five types: tRF-1, tRF-2, tRF-3, tRF-5, and inter tRF (i-tRF) [36,38]. Specifically, tRF-1 is derived from the 3′ tail of pre-tRNA and cleaved by RNaseZ or ELAC2; tRF-3 is generated by cleavage at a specific site in the T loop by Dicer, ANG, or members of the RNase A superfamily; tRF-5 originates from the 5′ end of mature tRNA and is cleaved by Dicer [[38], [39], [40]]. tRFs play crucial roles in cellular activities, including RNA silencing, translation inhibition, stress response, gene expression, cell cycle, and epigenetic regulation [41]. For example, tRF-3 enhances the translation efficiency of ribosomal protein S28 mRNA, while tRF-5 regulates the translation initiation process through abnormal modification [36,42]. Additionally, tRF-2 acts as a tumor suppressor by disrupting the interaction between YBX1 and oncogenic transcripts, thereby inhibiting cell proliferation and metastasis [43,44].

### 3.2 tRNA-derived stress-induced RNA (tiRNA)

tiRNA is a special type of small RNA primarily produced by angiogenin (ANG), which specifically cleaves the anticodon loop of mature tRNA to form fragments approximately 31–40 bases in length [45](Fig. 2B). The production of tiRNAs is usually triggered by various stress stimuli such as heat shock, cold shock, hypoxia, and oxidative stress [15]. Although ANG is the main catalytic enzyme, other enzymes like RNase T2 (RNY1) family, RNase L, endonuclease V, RNase I, and Schlafen13 (SFLN13) also participate in tiRNA formation under certain conditions [39]. tiRNAs are classified into two subtypes based on their source: tiRNA-5, about 30–35 nucleotides long, originates from the 5′ end of mature tRNA and extends to the anticodon loop; tiRNA-3, about 40–50 nucleotides long, extends from the 3′ end of mature tRNA to the anticodon loop. Notably, the expression pattern of tiRNAs does not directly correlate with the levels of their cognate tRNAs, suggesting specific regulatory functions rather than merely being degradation products of tRNAs [[46], [47], [48]]. tiRNAs are involved in various biological processes, such as ribosome competition and immune signal transduction, indicating roles in immune signaling due to the stable presence of tiRNA-5 in the blood [47]. They also exhibit anti-apoptotic functions by forming ribonucleoprotein complexes with cytochrome *c* (Cyt-C) released from mitochondria, thereby inhibiting apoptotic cell formation and activity [47]. Additionally, tiRNA-5 participates in translation inhibition by binding to the cold shock domain (CSD) of Y-box binding protein 1 (YBX-1), replacing eIF4F of mRNA, and promoting the formation of stress granules [48].

### 3.3 Sex hormone-dependent tRNA-Derived RNA (SHOT-RNA)

In addition to tiRNA-5 and tiRNA-3, there exists a unique category called SHOT-RNA. SHOT-RNAs are regulated by sex hormones and their receptors and are further categorized into 5′-SHOT-RNA and 3′-SHOT-RNA. Notably, 5′-SHOT-RNA contains a cyclic phosphate (cP) and an amino acid at the 3′ end, which is closely related to cell proliferation [49]. These findings have significantly expanded our understanding of tiRNAs and their biological functions.

## 4 Mechanism of action of tsRNA in pancreatic diseases

**Fig. 3:**
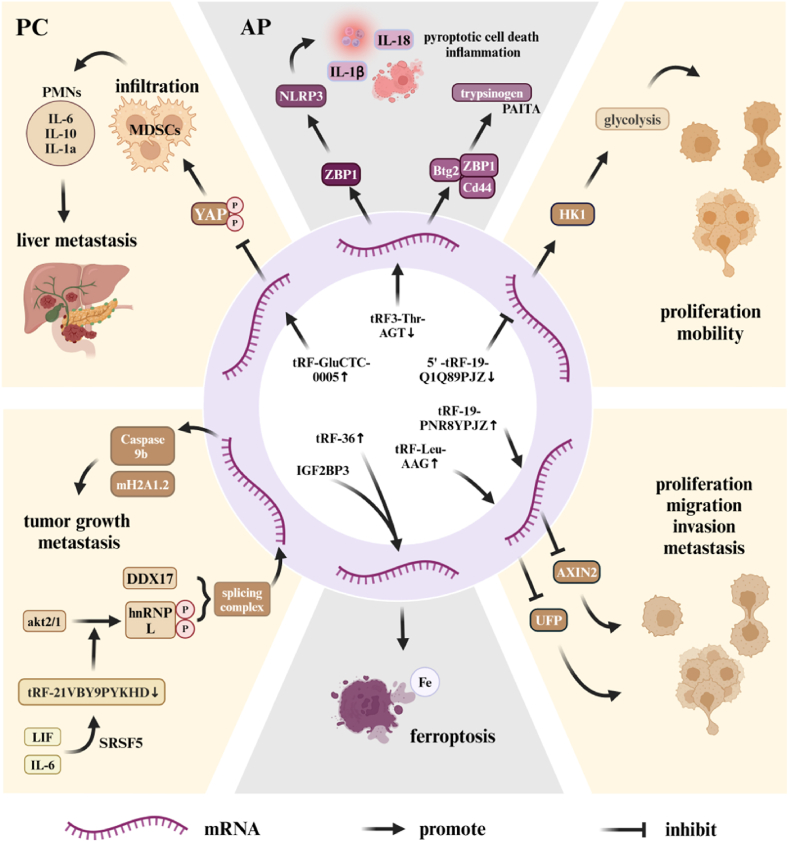
The Mechanism of Action of tsRNAs in Pancreatic Disease. The figure above highlights the differences between these two conditions: the four light yellow areas represent pancreatic cancer (PC), while the two light purple areas represent acute pancreatitis (AP). tsRNAs primarily influence the progression of pancreatic disease by modulating mRNA transcription and regulating the expression of genes such as HK1, ZBP1, Btg2, Cd44, AXIN2, and UPF1. tsRNA, Transfer RNA-derived small RNA; YAP, Yes-associated protein; PMNs, pre-metastatic niches; DDX17, dead-box helicase 17; hnRNPL, heterogeneous nuclear ribonucleoprotein L; IL-6, Interleukin 6; SRSF-5, serine- and arginine-rich splicing factor 5; UPF, upstream frameshift; HK1, hexokinase 1; ZBP1, Z-DNA-binding protein 1; NLRP3, NOD-like receptor protein 3; Btg2, B-cell translocation gene 2.

Extensive research has gradually revealed the pivotal role and clinical potential of tRNA-derived small RNAs (tsRNAs) in various diseases. We systematically summarized the expression profiles of various tsRNAs in pancreatic cancer and acute pancreatitis, along with their mechanisms of action, as shown in Table 1. Differential expression patterns were observed, with certain tsRNAs exhibiting upregulation or downregulation in specific conditions. For instance, tRF-36 is upregulated in AP [50], while tRF-Leu-AAG [51] and tRF-19-PNR8YPJZ [52] are upregulated in PC. Similarly, tRF-GluCTC-0005 shows increased expression in PDAC [53]. Conversely, several tsRNAs are downregulated, such as tRF3-Thr-AGT in AP [54,55], 5′-tRF-19-Q1Q89PJZ in PC [56], and tRF-21VBY9PYKHD in PDAC [57]. As detailed in Table 2 and illustrated in Fig. 3, these differentially expressed tsRNAs influence cellular behaviors in both in vitro and in vivo models through diverse mechanisms, including the regulation of protein-coding genes and participation in various signaling pathways. Their expression levels are closely associated with clinical pathological characteristics and disease prognosis, highlighting their potential significance in understanding and managing these conditions.

**Table 1:** Mechanisms of tsRNA Action in Pancreatic Diseases.

Disease	tsRNA	Expression	Upstream	Target	Downstream	Pathway
AP	tRF3-Thr-AGT [54]	downregulated	–	ZBP1	NLRP3	ZBP1/NLRP3
AP	tRF-36 [50]	upregulated	–	IGF2BP3	p53	Hippo
AP	tRF3-THr-AGT [55]	downregulated	–	Btg2, Cd44, Zbp1	–	–
PC	tRF-Leu-AAG [51]	upregulated	–	UPF1	–	GnRH
PC	tRF-19-PNR8YPJZ [52]	upregulated	–	AXIN2	–	Wnt
PC	5′-tRF-19-Q1Q89PJZ [56]	downregulated	AGO1, AGO3	HK1	–	Glycolysis/Gluconeogenesis
PDAC	tRF-GluCTC-0005 [53]	upregulated	–	WDRl	YAP	Hippo
PDAC	tRF-21VBY9PYKHD [57]	downregulated	SRSF5	hnRNRPL	Caspase9b, mH2A1.2	AKT

AP, acute pancreatitis; PC, Pancreatic cancer; PDAC, pancreatic ductal adenocarcinoma; ZBP1, Z-DNA-binding protein 1; NLRP3, NOD-like receptor protein 3; IGF2, insulin-like growth factor 2; BP3, binding protein 3; UPF1, upstream frameshift mutant 1; HK1, hexokinase 1; WDR1, WD repeat-containing protein 1; YAP, Yes-associated protein; SRSF5, serine- and arginine-rich splicing factor 5; hnRNRPL, heterogeneous nuclear ribonucleoprotein L; Btg2, B-cell translocation gene 2.

**Table 2:** Role and Clinical Value of tsRNAs in Pancreatic Diseases (In Vivo and In Vitro).

Disease	tsRNA	Effect in vitro/in vivo	Clinicopathological characteristic and Prognosis
AP	tRF3-Thr-AGT [54]	pyroptotic cell death↑ and inflammation↑/——	–
AP	tRF-36 [50]	ferroptosis↑/ferritin↑	–
AP	tRF3-THr-AGT [55]	PAITA↑/——	–
PC	tRF-Leu-AAG [51]	proliferation↑, migration↑, and invasion↑/——	–
PC	tRF-19-PNR8YPJZ [52]	proliferation↑, migration↑, and invasion↑/proliferation↑ and metastasis↑	associated with advanced clinical stage, poor survival and poor prognosis
PC	5′-tRF-19-Q1Q89PJZ [56]	glycolysis↑, proliferation↑, and mobility↑/proliferation↑ and metastasis↑	associated with advanced clinical characteristics and poor prognosis
PDAC	tRF-GluCTC-0005 [53]	metastasis↑ and infiltration↑/liver fibrosis↑	associated with shorter OS and DFS
PDAC	tRF-21VBY9PYKHD [57]	proliferation↑, invasion↑, apoptosis↓, migration↑, and colonization ability↑/tumor growth rate↑	associated with shorter survival time and poor prognosis

AP, acute pancreatitis; PC, Pancreatic cancer; PDAC, pancreatic ductal adenocarcinoma; PAITA, pancreatic acinar intracellular trypsinogen activation; OS, overall survival; DFS, disease-free survival.

**Fig. 4:**
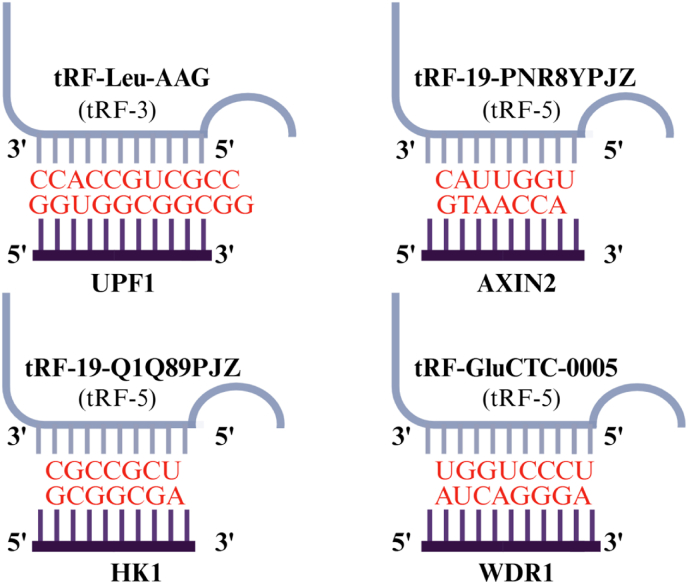
The binding sites of tsRNAs and Pancreatic cancer related PCGs. tsRNA, Transfer RNA-derived small RNA; PCGs, protein-coding genes.

In PC, the upregulation of tRF-Leu-AAG promotes cancer cell proliferation, migration, and invasion, while its downregulation inhibits these abilities. Studies have shown that UPF1, a downstream target gene of tRF-Leu-AAG, promotes the development of pancreatic cancer when downregulated [51]. Similarly, tRF-19-PNR8YPJZ promotes pancreatic cancer migration and invasion by acting through the AXIN2 axis [52] (Table 1, Table 2, Fig. 3). In PDAC, the upregulation of tRF-GluCTC-0005 enhances the mRNA stability of WDR1, thereby promoting cancer cell proliferation, migration, and invasion [58], and also plays a crucial role in PDAC liver metastasis [53]. On the other hand, the downregulation of tRF-19-Q1Q89PJZ in pancreatic cancer promotes cell proliferation and metastasis by inhibiting HK1 expression [56]. tRF-21-VBY9PYKHD (tRF-21) acts as a tumor suppressor in PDAC, with its downregulation promoting cancer cell proliferation, migration, and invasion. This tsRNA is involved in the AKT2/1-mediated hnRNP L phosphorylation process, interacts with DDX17, forms Caspase 9b and mH2A1.2, and promotes the malignant phenotype of PDAC cells [57] (Table 1, Table 2). Additionally, tRF-Leu-AAG, tRF-19-PNR8YPJZ, tRF-19-Q1Q89PJZ, and tRF-GluCTC-0005 have been confirmed as regulators of protein-coding genes (PCGs), with their binding sites illustrated in Fig. 4.

In AP, the expression of tRF-36 is significantly increased, promoting pancreatic follicular cell ferroptosis. This mechanism involves interaction with IGF2BP3, which guides to the m6A modification site of p53 mRNA, enhancing p53 mRNA stability and supporting ferroptosis [50,59,60]. In contrast, tRF3-Thr-AGT is downregulated in AP, promoting cell pyroptosis and inflammation by affecting NLRP3-mediated pyroptosis and inflammatory processes through the regulation of ZBP1. This reveals its potential role in protecting acinar cells [54,61]. Additionally, the downregulation of tRF3-Thr-AGT is associated with PAITA, which may lead to increased levels of trypsinogen activation, aggravating the pathological process of AP [55] (Table 1, Table 2, Fig. 3).

## 5 tsRNA as a biomarker for clinical diagnosis and prognosis of pancreatic cancer

The detection of tsRNA biomarkers primarily involves two steps: expression profile analysis and functional enrichment analysis, which are crucial for revealing their biological significance [62]. This process utilizes various tools and databases such as tRFdb, MINTbase, and tDRmapper (Table 3) to support the identification and analysis of tsRNA. To verify tsRNA expression, technologies like qRT-PCR/Real-time PCR are often employed to ensure accurate results. Additionally, platforms like DAVID, GO, and KEGG (Table 4) are used to predict tsRNA targets, providing insights into their functions. tsRNAs show great potential in disease diagnosis and treatment, particularly in pancreatic cancer, where tsRNA-MetCAT-37 and tsRNA-ValTAC-41 have emerged as potential diagnostic or prognostic biomarkers, offering a new perspective for precision medicine in pancreatic cancer.

**Table 3:** tsRNA Database Overview.

Name	Website	Comment
tRFdb [63]	http://genome.bioch.virginia.edu/trfdb/	The first tRF database contains sequences and read counts of three classes of tRFs from eight species.
MINTbase [16]	http://cm.jefferson.edu/MINTbase/	A web-based framework serves a dual purpose as a repository of tRF content and a tool for the interactive exploration of these newly discovered molecules.
tDRmapper [64]	https://github.com/sararselitsky/tDRmapper	A tool provides standardized nomenclature and quantification schemes and includes graphical visualizations to facilitate the discovery of novel tRNA and tRNA-derived RNA biology.
tRF2Cancer [17]	http://rna.sysu.edu.cn/tRFfinder/	A web-based integrated computational system accurately identifies tRFs from sRNA deep sequencing data and assesses their expression in multiple cancers.
MINTmap [65]	https://github.com/TJU-CMC-Org/MINTmap/	A tool is specifically developed for rapid, definitive, and exhaustive identification of tRFs in short RNA-seq datasets, explicitly calculating and reporting the raw and normalized abundance of discovered tRFs.
tRFexplorer [66]	https://trfexplorer.cloud	A tool provides users with expression profiles of each tRNA-derived noncoding RNA in every NCI-60 cell line and for every TCGA tumor type.
OncotRF [66]	http://bioinformatics.zju.edu.cn/OncotRF	A tool enables the identification of differentially expressed tRFs, prediction of their functions, and identification of diagnostic and prognostic biomarkers in cancer.
tsRBase [67]	http://www.tsrbase.org	A database integrates the expression patterns and functional information of tsRNAs in multiple species, describing specific expression patterns of tsRNAs under different conditions, making it the most comprehensive and systematic tsRNA repository.
tRFtarget [67]	http://trftarget.net	A database for transcriptome-wide tRF target prediction allows queries about the interactions between tRFs and transcripts in eight species, promoting the study of tRF molecular functions and mechanisms.
tsRFun [68]	http://biomed.nscc-gz.cn/DB/tsRFun/	A systematic and comprehensive tsRNA platform provides data resources and multiple analytical tools for studying known and novel tsRNAs and predicting their functions.

**Table 4:** Bioinformatics analysis of tsRNAs in Pancreatic cancer.

tsRNAs	Methods for Expression profile analysis	Methods for Functional enrichment analysis	Ref
AS-tDR-000064 AS-tDR-000069 AS-tDR-000102 AS-tDR-001391	Genomic tRNA Database and sequencing/qRT-PCR	DAVID databases, GO, KEGG, miRanda and TargetScan	[7]
tsRNA-MetCAT-37 tsRNA-ValTAC-41	miRBase database, piwi interacting RNA (piRNA) database, NCBI, Genomic tRNA database, tRFdb, MintBase and sequencing/Real-time PCR	GO, KEGG, miRanda and RNAhybrid	[69]
tRF-Pro-AGG-004 tRF-Leu-CAG-002	Sequencing/qRT-PCR	GO, KEGG and RNAhybrid	[70]
tRF3-Thr-AGT	GtRNAdb and Sequencing/Real-time PCR	DAVID databases, GO, KEGG and miRanda	[55]

NCBI, the National Center for Biotechnology Information; DAVID, Database for Annotation, Visualization and Integrated Discovery; GO, Gene Ontology; KEGG, Kyoto Encyclopedia of Genes and Genomes.

### 5.1 Diagnostic biomarkers

In the serum of patients with pancreatic ductal adenocarcinoma (PDAC), the expression of tsRNA-MetCAT-37, tsRNA-ValTAC-41, and tsRNA-ThrTGT-23 was significantly increased (Table 5). ROC analysis showed that the AUC values of tsRNA-MetCAT-37 and tsRNA-ValTAC-41 were 0.687 and 0.793, respectively, indicating good diagnostic potential. Compared to the traditional diagnostic marker CA19-9 (AUC = 0.906, sensitivity 85.9 %, specificity 97.0 %), combining tsRNA-MetCAT-37 or tsRNA-ValTAC-41 with CA19-9 increased the AUC to 0.949 and 0.947, respectively, with sensitivities increasing to 87.8 % and 90.2 %, underscoring their value as diagnostic biomarkers for PDAC [69].

**Table 5:** Potential tsRNA Biomarkers in Pancreatic Cancer.

Type	Referece Biomarker	tsRNA	Clinical application	Ref
PDAC (tissues)	CA19-9:AUC = 0.585; CEA:AUC = 0.583.	tRF-Pro-CGG:AUC = 0.92, SEN = 0.757, SPE = 0.933.	prognosis	[71]
PDAC (serum)	CA19-9:AUC = 0.906, SEN = 0.859, SPE = 0.97.	tsRNA-MetCAT-37:AUC = 0.687.	diagnosis	[69]
		tsRNA-Val-TAC-41:AUC = 0.793.	diagnosis	
		CA19-9+tsRNA-MetCAT-37:AUC = 0.949, SEN = 0.878.	–	
		CA19-9+tsRNA-Val-TAC-41:AUC = 0.947, SEN = 0.902.	–	
PC (serum)	CA19-9:SEN = 0.635, SPE = 0.596; CEA:SEN = 0.631, SPE = 0.585.	tRF-Leu-CAG-002:AUC = 0.78, SEN = 0.64, SPE = 0.772.	prognosis and diagnosis	[69]
		tRF-Pro-AGG-004:AUC = 0.9, SEN = 0.725, SPE = 0.988.	prognosis and diagnosis	
		tRF-Leu-CAG-002+tRF-Pro-AGG-004:AUC = 0.94, SEN = 0.85, SPE = 0.964.	–	
PC∗ (serum)		tRF-Leu-CAG-002+tRF-Pro-AGG-004:AUC = 0.84, SEN = 0.75, SPE = 0.83.	–	

PDAC, pancreatic ductal adenocarcinoma; PC, pancreatic cancer; PC∗, early PC (stage I and II); CA19-9, carbohydrate antigen 19-9; TNM, tumor-node-metastasis; OS, overall survival; CEA, carcinoembryonic antigen.

Additionally, the expression of tRF-Pro-AGG-004 and tRF-Leu-CAG-002 was significantly increased in the serum of pancreatic cancer patients (Table 5). tRF-Pro-AGG-004 was primarily present in the exosome-free supernatant, while tRF-Leu-CAG-002 was mainly enriched in exosomes. ROC analysis revealed AUC values of 0.90 and 0.78 for tRF-Pro-AGG-004 and tRF-Leu-CAG-002, respectively, with sensitivities of 72.5 % and 64 %, and specificities of 98.8 % and 77.2 %, demonstrating their accuracy in early pancreatic cancer diagnosis [70]. Notably, when these two tsRNAs were used in combination, the AUC value reached 0.84, with a sensitivity of 75.0 % and a specificity of 83.0 %, surpassing commonly used clinical markers CA19-9 and CEA, revealing their significant potential as early-stage disease biomarkers [70].

### 5.2 Prognostic biomarkers

In PDAC tissues, tRF-Pro-CGG expression was significantly downregulated and primarily localized in the cytoplasm of PDAC cells (Table 5). This expression difference was closely related to TNM staging and lymph node metastasis, being significantly correlated with advanced PDAC. The expression of tRF-Pro-CGG was positively correlated with patient overall survival (OS), with a median OS of 7 months in the low expression group and 31 months in the high expression group. ROC analysis indicated an AUC of 0.92, with sensitivity and specificity of 75.7 % and 93.3 %, respectively [71], highlighting its potential as a PDAC biomarker.

In the serum of PDAC patients, tsRNA-MetCAT-37, tsRNA-ValTAC-41, and tsRNA-ThrTGT-23 were upregulated (Table 5). Patients with low serum tsRNA-ValTAC-41 levels had better OS, and its expression was higher in locally advanced and metastatic PDAC [69], suggesting its potential as a prognostic biomarker.

Elevated levels of tRF-Pro-AGG-004 and tRF-Leu-CAG-002 in the serum of pancreatic cancer patients were positively correlated with tumor weight (Table 5). Patients with high expression of these tsRNAs had poor prognosis and shorter survival times [70], indicating their potential as prognostic markers. Other potential biomarkers in pancreatic cancer include AS-tDR-000064, AS-tDR-000069, AS-tDR-000102, and AS-tDR-001391, whose expression levels are either upregulated or downregulated. Bioinformatics analysis showed that their target genes are associated with different signaling pathways [7], providing new directions for pancreatic cancer research.

## 6 The role of tsRNA in pancreatic cancer treatment

Studies have revealed that abnormally expressed transfer RNA (tsRNA) plays a key role in the progression of pancreatic cancer (PC) and holds promise as a target for cancer treatment. In PC tissues, tRF-Leu-AAG is significantly upregulated and may promote PC cell proliferation and migration by regulating UPF1, making it a potential target for PC treatment [51]. Plasma sample comparisons found that 5′-tRF-19-Q1Q89PJZ is downregulated in PC and associated with poor prognosis. It inhibits HK1 expression, thereby reducing PC cell glycolysis, proliferation, and migration, making it a key candidate target for PC treatment [56].

In pancreatic ductal adenocarcinoma (PDAC), tRF-21VBY9PYKHD is downregulated and linked to poor prognosis. It inhibits the malignant phenotype of PDAC cells by promoting hnRNP L phosphorylation and has potential as a PDAC therapeutic agent [57]. Additionally, tRF-19-PNR8YPJZ is upregulated in PC tissues and associated with poor prognosis. It promotes PC cell migration and invasion through the AXIN2 axis, marking it as an important target for PC treatment [52]. In early liver metastasis of PDAC, tRF-GluCTC-0005 is upregulated in serum exosomes and associated with shortened overall survival (OS) and disease-free survival (DFS). It promotes tumor progression and liver metastasis by binding to the 3′ untranslated region of WDR1 mRNA, suggesting it as a new target for pancreatic cancer treatment [58].

tsRNAs also play a central role in the formation of drug resistance in various cancers. For example, exosome-delivered tRF-16-K8J7K1B significantly enhances Tamoxifen resistance in breast cancer by inhibiting drug-induced cell apoptosis [72]. However, the specific association between tsRNA and drug resistance in pancreatic cancer (PC) remains unclear. To further explore this relationship, the CADDIE database was used to analyze the downstream targets of PC-related tsRNAs, leading to the identification of drugs such as Lonidamine that can target tsRNA-related HK1 proteins.

Although tsRNA is just one part of the complex mechanism network underlying cancer development, targeting strategies for tsRNA have shown significant potential in reducing drug resistance and improving treatment outcomes. Therefore, in-depth research into the mechanisms of tsRNA in PC resistance and the development of corresponding targeted drugs are expected to provide new and effective strategies for treating pancreatic cancer.

## 7 Current research and future directions

As scientific research deepens, the regulatory mechanisms of tsRNAs in pancreatic cancer (PC) and acute pancreatitis (AP) are gradually becoming clearer. However, further exploration is needed to fully understand their clinical value in pancreatic diseases and potential as therapeutic targets. Compared to other systemic diseases, the research on the regulatory mechanisms of tsRNAs in PC and AP remains insufficient, indicating a vast area for future investigation.

tsRNAs exhibit unique effects in various disease pathways. In AP, tRF3-Thr-AGT promotes cytotoxic cell death and inflammatory responses by regulating the ZBP1/NLRP3 pathway [54]. Similarly, tRF-36 plays a key role in pancreatic follicular cell ferroptosis and cell metabolism [50].

In pancreatic cancer, particularly in pancreatic ductal adenocarcinoma (PDAC), tsRNAs have a significant impact. For instance, tRF-Pro-CGG inhibits PDAC growth and metastasis through the P13K/protein kinase-B signaling pathway [71]. The target gene SRSF5 of tRF-21VBY9PYKHD is involved in the AKT pathway, promoting PDAC proliferation and invasion while inhibiting apoptosis [57]. Both tRF-Leu-CAG-002 and tRF-Pro-AGG-004 enhance PC cell proliferation and invasion via the Hippo pathway [70]. Additionally, 5′-tRF-19-Q1Q89PJZ supports PC cell proliferation, migration, and glycolysis through the glycolysis/gluconeogenesis signaling pathway [73]. The target gene WDR1 of tRF-GluCTC-0005 activates the Hippo signaling pathway, promoting PDAC liver metastasis [53]. tRF-19-PNR8YPJZ facilitates PC cell proliferation and metastasis through the Wnt pathway [52].

Beyond pancreatic diseases, tsRNAs also play crucial roles in other pathways. For example, they influence gastric cancer (GC) progression through the MAPK pathway [[74], [75], [76], [77]] and diabetic retinopathy (DR) progression through the HIF-1 pathway [45,78]. Understanding these pathways in relation to specific tsRNAs will enhance our comprehension of disease mechanisms.

Despite promising initial results, tsRNA research is still in its early stages and faces several challenges. These include the need for improved annotation tools and databases, deeper exploration of tsRNA mechanisms, and overcoming the complexities of tsRNA detection, classification, and inconsistent naming conventions. Addressing these challenges requires sustained effort and innovation to achieve significant breakthroughs in the field of tsRNA research.

## 8 Conclusion

In summary, this study provides a comprehensive and systematic exploration of the mechanisms of action of tsRNAs in pancreatic diseases. It delves deeply into their potential as biomarkers and therapeutic targets, offering valuable insights and guidance for future research. Despite these advances, current research still has its limitations. Looking ahead, it is crucial to focus on the application of tsRNAs related to pancreatic diseases in early diagnosis, prognosis assessment, and treatment strategies. This will enable a more comprehensive understanding of their role in tumor development and pave the way for more precise and effective treatment options for patients. Continued efforts in this direction are essential to fully realize the clinical potential of tsRNAs in pancreatic diseases.

## CRediT authorship contribution statement

**Yan Pan:** Writing – review & editing, Methodology, Funding acquisition, Data curation. **Xiaowei Ying:** Writing – review & editing, Writing – original draft, Visualization, Methodology. **Xueting Zhang:** Writing – original draft, Visualization. **Hongting Jiang:** Writing – original draft, Visualization. **Junjie Yan:** Writing – original draft, Visualization. **Shiwei Duan:** Writing – review & editing, Methodology, Investigation, Funding acquisition.

## Ethics approval and consent to participate

Not applicable.

## Consent for publication

All authors have read and agreed to the published version of the manuscript.

## Availability of data and material

Not applicable.

## Competing interests

All authors declare no competing interests.

## Declaration of interests

All authors declare no competing interests.

## Funding

This study was supported by the Qiantang Scholars Fund in Hangzhou City University (No. 210000–581835), Zhejiang Province Traditional Chinese Medicine Science and Technology Project (2022ZQ068).

## Conflict of interest statement

No potential conflict of interest was reported by the authors.
